# Reducing endoscopic procedure backlog by improving efficiency: a predictive model and machine learning-based scheduling approach

**DOI:** 10.1093/jcag/gwaf006

**Published:** 2026-02-05

**Authors:** Tu-San Pham, Héloïse Gachet, Waleed Aljohani, Jeanne Archambault, Myriam Martel, Alan Barkun, Louis-Martin Rousseau

**Affiliations:** Polytechnique Montreal, Montréal, QC, H3T 1J4, Canada; Ecole des Ponts ParisTech, France; Research Institute of the McGill University Health Center, Montréal, QC, Canada; Ecole des Ponts ParisTech, France; Research Institute of the McGill University Health Center, Montréal, QC, Canada; Gastroenterology, McGill University, Montréal, QC, Canada; Polytechnique Montreal, Montréal, QC, H3T 1J4, Canada

**Keywords:** healthcare, machine learning, predictive model, scheduling

## Abstract

**Background:**

The COVID-19 pandemic led to a significant decrease in endoscopic procedure volumes, resulting in a backlog of patients awaiting investigation. Our study thus aimed to develop a machine learning-based scheduling tool to improve resource utilization, enhance system efficiency, and increase patient throughput, ultimately reducing procedural delays.

**Methods:**

In the first phase, machine learning methods were applied to historical data to predict procedure duration based on patient characteristics and environmental factors. In the second phase, a scheduling module was built using a greedy heuristic and a Mixed Integer Programming (MIP) model to optimize resource utilization.

**Results:**

We showed that among the tested models, an XGBoost regression model was selected with a mean absolute error of 5.67 minutes on the test set. The simulation results demonstrated that MIP increased the number of patients scheduled by 5.9% while reducing mean waiting time from 19.5 days to 17.3 days over a waiting list of 1,000 patients, evaluated within a 2-week period (10 working days). Simulations using real patient data showed that the MIP scheduled 8 more patients than the baseline. Numerical results confirmed higher resource utilization rates in adaptive schedules.

**Conclusions:**

Our study highlights the potential of a machine learning-based scheduling tool to enhance resource allocation, thus helping address backlogs in endoscopic procedures. Real-world clinical validation is now necessary to substantiate the tool’s effectiveness. Future work should prioritize prospective data collection to refine the predictive model and seamlessly integrate the tool into clinical workflows, ensuring its practical utility and success.

## Introduction

Gastrointestinal (GI) endoscopic procedures are amongst the most frequently practiced procedures in Canadian stand-alone clinics and hospitals with almost 2,000,000 procedures performed yearly.^1^ This activity plays a critical role in managing patients with digestive disorders, including colonoscopies that have contributed to decreasing the incidence and mortality of colorectal cancer, the 3rd most common malignancy in Canada.^2^ The COVID-19 pandemic and its mitigation practices brought about dramatic drops in endoscopic procedural volumes across the country,^3^ and globally.^4^ This is especially true for diagnostic procedures, with a great risk of many patients not being diagnosed in a timely manner with cancers, digestive and other.^5,6^ Wait times for endoscopic procedures, especially colonoscopy, that had already been an issue due to limited resources country-wide,^7^ have been lengthened even more dramatically since the pandemic. Modeling has suggested that the backlog in colonoscopies induced by COVID-related delays, unless tackled aggressively, could result in excess mortality due to colon cancer for many 10 years to come.^8^

Currently, most procedures are assigned a flat time slot of half-hour, except when doctors request longer procedural time allocation for selected cases. In practice, the actual procedure lengths vary greatly from less than 5 minutes to 40 minutes or more. Therefore, a flat-scheduling approach might not fully exploit medical resources and leave idle times between procedures. Resource utilization may be optimized by scheduling patients based on a precise prediction of each individual’s procedure length.

In this study, we provide a machine learning-based solution to increase patient throughput to reduce the backlog of patients awaiting procedures.

## Materials and methods

The project consisted of two components: predicting and scheduling. To develop a forecasting model, we adopted the well-defined CRISP-DM process (CRoss Industry Standard Process for Data Mining).^9^ For the scheduling component, we devised two strategies, using a greedy heuristic approach and a Mixed Integer Programming (MIP) model.

### Building a predictive model

The CRISP-DM process, which is depicted in Supplementary Figure 1, comprises of six distinct phases. In this paper, we outline the main steps involved in the process.

#### Business understanding

The project is conducted using data from the digestive endos-copy units of the McGill University Health Center (MUHC) at two sites: the Montreal General (MGH) and Royal Victoria Hospitals (RVH).

The MUHC has 6 digestive endoscopy rooms dedicated to performing elective procedures. Currently, all patients are given a fixed-length appointment of 30 minutes. After registration, the patient waits in a designated area before moving to a room for nurse assessment and preparation. The patient then enters the endoscopy room, where the doctor secures patient consent, explains the procedure, discusses risks, benefits, and alternatives. After the procedure, the patient is transferred to a recovery room. During the process, a time stamp was recorded at each critical step on a paper sheet by an attending nurse. To collect the data, we transferred the information onto a digital format platform. As the process is highly time intensive and prone to human error due to difficult reading and/or missing information on the sheet, the number of observations collected was limited.

To schedule a colonoscopy appointment, it is ideal to predict the total duration that a patient spends in the colonos-copy room. However, this information was either imprecise or missing in the majority of our data entries. Therefore, we predicted a procedural duration, which reflected the duration between the start and end of an actual digestive endoscopy. To schedule the appointment, a buffer time of 15 minutes for discussion, and for preparation/transferring the patient into and out of the endoscopy room was then added to the procedure duration.

#### Data understanding

The data on endoscopy procedures at the MUHC was gathered retrospectively over a period of 48 working days. The data were collected over two time frames: From March 18th, 2019 to April 23rd, 2019 (667 patients), and from January 3rd to February 5th, 2020 (665 patients). This resulted in a total of 1332 appointments for 7 different procedure types.

For each appointment, various types of information were recorded, such as patient age, sex, body mass index (BMI), medical history, procedure type, and indication. The list of features and their characteristics can be found in Table 1. Each appointment was treated as a separate data entry, with the procedure duration serving as its label. The shortest procedure in the dataset took 2 minutes while the longest lasted 1 hour and 25 minutes. On average, the procedure duration was 16.2 minutes with a standard deviation of 9.85 minutes. The distribution of procedure times in our dataset can be viewed in Supplementary Figure 2 (appendix).

**Table 1.** List of features

	**Feature**	**Type**	**Values/range and statistics**	**Note**
1	Sex	Boolean	51.2% male	Colonoscopy can be more challenging in women because the female pelvis is wider and deeper than the male pelvis [14]
2	Age	Categorical	<45 (17.8%), 45-59 (27%), 60 to 80 (47.1%), >80 (8%)	
3	BMI	Categorical	<25 (23.3%), from 25 to 30 (21.5%), >30 (12.5%), no information (42.7%)	
4	Patient priority	Categorical	Details in Table 2	
5	Comorbidity score	Categorical	0 (54.6%), 1 (22.7%), 2 (14.4%), 3 (6%), 4 (2.3%)	Colonoscopies are affected by a patient’s respiratory or cardiovascular conditions, leading to more complications [15]
6	Respiratory	Boolean		
7	Cardiovascular diseases	Boolean		
8	Diabetes	Boolean		
9	Former pelvic surgeries for females	Boolean	13.7% of patients had undergone previous pelvic surgery	In female patients, past pelvic surgeries can lead to technically more difficult (and lengthy) procedures and/or discomfort for the patients. [16].
10	Past failed colonoscopy	Boolean	1: patient has a failed colonoscopy in the past, 0: otherwise	
11	Reason of failed colonoscopy	Categorical	1: poor preparation, 2: technical difficulties, 3: disease-related factors, 4: others	
12	Procedure type	Categorical	Colonoscopy (64.2%), gastroscopy (30.7%), others (5.1%, including EUS Radial, EUS Linear, Sigmoidoscopy, Enteroscopy, Ileoscopy)	
13	Procedure indication	Categorical	High, intermediate, low importance, and surveillance	Procedure indications determine the patient’s priority^1^
14	Endoscopist	Categorical	Gastrointestinal (79:9%), colorectal (15:6%), hepatologic, and thoracic physicians	

In Quebec, a doctor referring a patient for a digestive endoscopy must provide indications on a province-wide colonoscopy referral sheet [17].

In Quebec, patients are classified into 5 categories based on their priority level.^10^ The distribution of patients by priority level in our dataset can be found in Supplementary Table 1 (appendix). Urgent patients *P*1 are not included in our analysis as they typically receive treatment in designated emergency rooms, intensive care units, or dedicated inpatient endoscopy suites (not included in this analysis).

#### Data preparation

Data cleaning: We cleaned the data by removing invalid and outlier appointments. Patients with missing procedure duration information were omitted. As a result, the dataset consisted of 1253 patients with complete information for building a predictive model.

Feature selection: We adopted a *wrapper method* to determine the most impactful features. A wrapper method is a feature selection process based on a specific machine learning algorithm to evaluate different combinations of features. We opted for backward elimination and used XGBoost Regressor as the machine learning algorithm. In backward elimination, the model was initially trained using all features, and the least important feature was identified via the importance plot computed on the training data. The model was retrained by dropping the least important feature. The process was iterated to identify the set of features that lead to the minimum validation error. To implement this approach, we utilized scikitlearn’s SequentialFeatureSelector, which systematically evaluates subsets of features based on the performance of a given estimator. The selection process optimizes a scoring criterion, which in our case was the mean absolute error (MAE).

#### Modelling

Various predictive models (including Linear Regression, Random Forest, Extreme Gradient Boosting (XGBoost), Gaussian Process (GP), and Multi-layer Perceptron (MLP)) were tested and compared. The detailed descriptions of those models are provided in the Appendix. To ensure unbiased evaluation, the dataset was randomly split into a training set and a testing set in an 80/20 ratio. This random splitting process preserves the overall distribution of characteristics across both sets, minimizing potential biases and ensuring that the training and testing cohorts are representative of the entire dataset. The training set of 1002 patients was used to train the models, which were then tested on the testing set of a separate 251 patients.

#### Scheduling patients based on a predictive model

Given a prediction of procedure duration, a schedule can be created by solving an optimization problem to maximize the number of procedures done in a given period. The goal of the scheduling phase was to optimize resource utilization and patient throughput. We proposed two scheduling policies, namely a greedy heuristic approach and a Mixed Integer Programming (MIP) model.

Greedy heuristic: The greedy heuristic involved creating a global waiting list that sorted patients based on three criteria: (1) descending order of priority; (2) ascending order of remaining time until their due dates; and finally (3) a descending order of their procedure duration. Patients were then scheduled in the order of the waiting list. If the patient already had a designated endoscopist, they were assigned to the earliest available time slot of that endoscopist. Otherwise, an endoscopist who accepts non-nominative patients was chosen from a list, based on their availability, and assigned to the patient.

MIP formulation: The problem involved scheduling endos-copy procedures for a set of patients P, given a set of working days *D* in the planning horizon, and a set of endoscopists E. Each working day of an endoscopist is divided into blocks, with each block consisting of consecutive time slots. The working block of an endoscopist typically lasts half a day (morning or afternoon, an endoscopist may also have 2 successive blocks on a given date—*i.e.* the whole day). The total working time of endoscopist *e* on day *d* is denoted as δ^d^  _e_.. Each patient *p* ∈ P has an admission date *a_p_*, on which their procedure request entered the waiting list and a recommended due date *d_p_* calculated based on their priority. Patient *p* requests a procedure with a duration of δ_*p*_ minutes, derived from the predictive model discussed above. Let E_*p*_ be the set of endoscopists that can perform the requested procedure of patient *p*. If the patient already has a designated endoscopist *p_e_*, then E *_p_* = {*p_e_*}.

We define a binary variable x^d^  _pe_ that holds value 1 if patient *p* is assigned to endoscopist *e* ∈ E on day *d*. The model is as follows:


(1) $$\omega {}_{1}\sum_{p\in P}^{}\sum_{e\in E}^{}\sum_{d\in D}^{}X_{pe}^{d}-\omega {}_{1}\sum_{p\in P}^{}\sum_{e\in E}^{}\sum_{d\in D,d>d{}_{p}}^{}(d-d{}_{p}){}^{2}x_{pe}^{d}$$

Subject to:


(2) $$x_{pe}^{d}=0,\forall p\in P,\forall d\in D,\forall e\in E\setminus E_{p}$$

(3) $$\sum_{p\in P}\delta_{p}\times x_{pe}^{d}\leq \delta_{e}^{d},\forall d\in D,\forall e\in E$$

(4) $$\sum_{e\in E_{p}}\sum_{d\in D}x_{pe}^{d}\leq 1,\forall p\in P$$

(5) $$x_{pe}^{d}\in \{0,1\},\forall p\in P,\forall e\in E,\forall d\in D$$

The objective function (1) maximizes the number of patients scheduled in the given planning horizon and minimizes the overdue times, with the respective weights ω_1_ and ω_2_. The values of the weights are chosen so that maximizing the total number of patients scheduled is prioritized over minimizing the total number of overdue days. The square penalty over the number of overdue days encourages a fair distribution of overdue time between patients. Constraints (2) prevent a patient from being assigned to a non-compatible endoscopist. Constraints (3) enforce the treatment capacity of endoscopists. Constraints (4) make sure that each patient has at most one appointment. Constraints (5) are domain constraints.

#### Simulation studies

Two experiments were conducted on two hospital sites, each with one procedural room, to evaluate the proposed approaches. The first experiment aimed to evaluate if the predicted procedure time could improve patient throughput compared to the default half-hour schedule. The second experiment aimed to analyze if scheduling using the predicted duration could lead to accumulated overtime in the schedules using real data collected from the MUHC. As the patients in our dataset are scattered over a wide range of dates with no complete set of patients arriving in a single day, we randomly sampled patients from the real patient pool to create hypothetical patients for the simulation. These patients were redistributed into the set of days within our simulation horizon to ensure all characteristics of the real patients were preserved. Patient procedural dates were generated within a range of [-7, 14] days from the first day of the planning period, and the procedure duration was predicted using a trained regression model. Three approaches were compared in each experiment: (1) the greedy heuristic with a flat appointment duration of half-hour, (2) the predictive-greedy approach with *predictive* appointment duration, and (3) the MIP approach. For each model, we reported the mean square error (MSE) and the mean absolute error (MAE) on the test set.

*Scheduling* We evaluated the scheduling performance on a waiting list of 1000 patients created from historical data. These 1000 patients were generated by randomly sampling the real data. The random sampling ensured that the distribution of all personal features (e.g., procedure type, sex, BMI, historical diseases, etc.) accurately reflected the original dataset’s distribution. Patient admission dates were generated so that their due dates fell within the range of [−7, 14] days from the first day of the planning period. After sampling, the hypothetical patients were assigned to a short list of endoscopists. We implemented all algorithms using Python and utilized IBM’s CPLEX v22.1 as the MIP solver. We scheduled appointments for a 10-day working period

## Results

**Figure 1.**
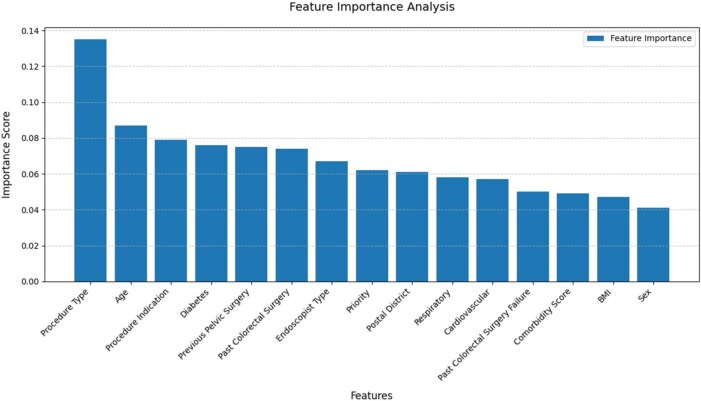
Feature Importance for XGBoost Regressor with all features

Predictive model Feature selection: Figure 1 shows an example of the important plot in the initial feature selection iteration where all features were included. The figure highlights that the procedure type had the greatest impact on the procedure length, while sex had the least effect. Consequently, sex was removed from the feature list, and the process was repeated until the minimal number of features remained.

The final list of features was determined by analyzing the plot presented in Supplementary Figure 3 (appendix), which illustrates the negative Mean Absolute Error (negative MAE—the higher the better) as a function of the number of features remaining at each iteration on both the training and test sets. The plot revealed that the MAE did not improve significantly with more than four features. Consequently, the decision was made to limit the number of features to four to prevent overfitting. The final list of features, ranked in order of importance, included procedure type, endoscopic type, the presence of co-morbidities including: respiratory diseases, and type 2 diabetes mellitus.

Comparison of models: The results given by all tested models are summarized in Supplementary Table 2. XGBoost resulted in the lowest MSE of 63.09. In addition, XGBoost model is also easy to interpret. Therefore, we chose XGBoost as the final predictive model.

### Scheduling results on generated patients

The scheduling results are summarized in Table 2.

**Table 2.** Results of the scheduling phase on a waiting list of 1000 patients. For each patient type, we report the number of patients scheduled and the average late time (in days).

	**All**		**P2**		**P3**		**P4**		**P5**		**SURV**	
	**#patients**	**avg**	**#patients**	**avg**	**#patients**	**avg**	**#patients**	**avg**	**#patients**	**avg**	**#patients**	**avg**
Fixed 30’	780	19.52	15	20.13	339	19.52	121	19.57	108	19.60	197	19.38
Predictive Greedy	781	19.51	15	20.13	340	19.50	120	19.56	110	19.55	196	19.40
MIP	826	17.33	12	12.67	280	13.45	147	17.00	131	21.33	256	19.93

**Table 3.** Results of the simulation on real data.

	**# patients**	**Avg. late (days)**	**Total overtime (minutes)**	**Avg. overtime (minutes/day/room)**	**Total earliness (minutes)**	**Avg. earliness (minutes/day/room)**
Fixed 30’	203	18.14	208	13.00	385	24.06
Greedy	204	18.11	238	14.88	235	14.69
MIP	211	16.39	321	20.06	156	9.75

With the available resources, we were able to schedule 780 out of 1000 patients on the waiting list using the default 30-minute appointment slots. The predictive-greedy heuristic, which used adaptive appointment durations, was able to schedule one additional patient compared to the baseline. The MIP approach outperformed the greedy heuristic by scheduling an additional 46 patients (5.9%) and reducing the mean waiting time from 19.5 days to approximately 17 days. Moreover, it successfully decreased the mean number of late days for patients who required higher priorities.

### Scheduling results on real data

We performed a simulation using real data to examine if inaccurate predictions could lead to overbooking and overworking of medical staff. The simulation was carried out over eight days, using a test set of 251 patients and a subset of endoscopists. The simulation results are summarized in Table  3, which reports the total number of patients scheduled and the mean number of late days per patient for each approach. Our resources included two procedure rooms, each with a daily operational time budget of 390 minutes (*i.e.* excluding personal break times), with overtime should the total appointment duration of patients scheduled exceeded this duration. The table also displays the total overtime incurred during the planning period, the mean overdue time per day per room, and the “earliness” time, which represents the difference between the time budget and the actual total appointment duration each day.

The fixed 30-minute appointment schedule could accommodate 203 patients, with a mean delay of 18.14 days per patient. Although the default schedule had an average earliness of 24 minutes per room per day, 208 minutes of overtime were still recorded during the planning period, equivalent to a mean of 13 minutes per day per room. Both the greedy heuristic and MIP approaches outperformed the baseline. The greedy heuristic scheduled one additional patient, with an overtime of 15 minutes per room per day, while MIP scheduled 8 more patients than the baseline with a mean overtime of 20 minutes per room per day. As for the earliness of each algorithm, the fixed 30-minute appointment schedule had 24 minutes of earliness per room per day, while the other approaches had lower numbers (14.69 minutes with the greedy heuristic and 9.75 minutes with MIP). The numerical results confirmed that resource utilization rates were higher in adaptive schedules (for more detailed results, please refer to Supplementary Table 3 (appendix)).

## Discussion

Our study represents the first published attempt to propose adaptive schedules that improve resource utilization and patient throughput in scheduling digestive endoscopy as a result of trying improve throughput in the post-pandemic era. By addressing the lack of a reliable and precise method for predicting endoscopy procedure time and by applying an optimization model, we strive to provide timely and efficient care to our patients.

Various predicted models were tested and compared using separate datasets for training and testing. The XGBoost model was chosen as final predictive model, as it resulted in the lowest MSE (63.09), with a mean absolute error of 5.67 minutes on the test set. The simulation results demonstrated superior results with the MIP model, outperforming the greedy heuristic approach, allowing to schedule an additional 46 patients (5.9%) while reducing the mean late time from 19.5 days to approximately 17 days. Simulations using real patient data showed that the MIP scheduled 8 more patients than the baseline with a mean overtime of 20 minutes per room per day, while minimizing was estimated at 9.75 minutes. The numerical results confirmed that resource utilization rates were higher in adaptive schedules.

Allowing for adapted allocation of endoscopy operative time requires two distinct tasks. The first task is to accurately predict the expected procedure duration for each patient. In the literature, few attempts have been made to analyse the procedure time of digestive endoscopic procedures. Jain et al.^11^ analysed the data collected and proposed a linear regression model to fit the data, but no information on the precision of the model was given. Some other studies^12^ examined the effect of age, sex, etc. on procedural time and level of procedural difficulty. A systematic and quantitative method for accurately predicting endoscopy procedure time is thus still lacking in the literature.

The second task involves defining and solving an optimization model based on the predicted procedure duration. Similar efforts have been made in other domains, such as surgery scheduling and patient scheduling in radiotherapy. For instance, Devi et al.^13^ developed a framework to forecast surgery time, which they used to create algorithms for scheduling operating rooms. Bentayeb et al.^14^ also used a service-time predictive model to improve patient scheduling at a radiotherapy center.

Although this optimization of scheduling exercise adopted state-of-the-art approaches adapted to the clinical scenarios tested, they are based on the practice at two of the busiest endoscopy unites in the province of Québec, although the case mix must of course be considered when looking the general-izability of the results.

In the future, the scheduling tool can be further improved by collecting prospective data to better calibrate the predictive model. Implementing such adapted scheduling models will require assessing the impact on staff resiliency and patient no-show rates.

## Conclusion

In this paper, we propose to predict endoscopic procedure durations based on historical data from two large-volume general endoscopy units. These predictions are then utilized in an optimization model to schedule patient appointments, evaluating both a greedy heuristic and a MIP model. Compared to a standard 30-minute slot assignment, the MIP model significantly reduces the average late days for patients compared to other approaches, a finding confirmed with real data simulation. Consequently, we conclude that our machine learning-based scheduling tool is proven effective in reducing backlog and enhancing patient satisfaction.

## Supplementary Material

gwaf006_Supplementary_Data

## Data Availability

The data underlying this article will be shared on reasonable request to the corresponding author.

## References

[1] Armstrong D , Barkun A, Bridges R, et al Canadian Association of Gastroenterology Safety and Quality Indicators in Endoscopy Consensus Group. Canadian Association of Gastroenterology consensus guidelines on safety and quality indicators in endoscopy. Can J Gastroenterol. 2012;26(1):17–31. 10.1155/2012/173739 22308578 PMC3275402

[2] Nishihara R , Wu K, Lochhead P, et al Long-term colorectal-cancer incidence and mortality after lower endoscopy. N Engl J Med. 2013;369(12):1095–1105. 10.1056/NEJMoa1301969 24047059 PMC3840160

[3] Menard C , Waschke K, Tse F, et al COVID-19: framework for the resumption of endoscopic activities from the Canadian Association of Gastroenterology. J. Can. Assoc. Gastroenterol.. 2020;3(5):243–245. 10.1093/jcag/gwaa016 32885139 PMC7337808

[4] Parasa S , Reddy N, Faigel DO, Repici A, Emura F, Sharma P. Global impact of the COVID-19 pandemic on endoscopy: an international survey of 252 centers from 55 countries. Gastroenterology. 2020;159(4):1579–1581.e5. 10.1053/j.gastro.2020.06.009 32534934 PMC7289081

[5] Corley DA , Jensen CD, Quinn VP, et al Association between time to colonoscopy after a positive fecal test result and risk of colorectal cancer and cancer stage at diagnosis. JAMA. 2017;317(16):1631–1641. 10.1001/jama.2017.3634 28444278 PMC6343838

[6] UK Government. (n.d.). Explanation of the updates to infection prevention and control guidance. GOV.UK. Retrieved 2024. https://www.gov.uk/government/publications/wuhan-novel-coronavirus-infection-prevention-and-control

[7] Leddin D , Armstrong D, Barkun AN, et al Access to specialist gastroenterology care in Canada: comparison of wait times and consensus targets. Can J Gastroenterol. 2008;22(2):161–167. 10.1155/2008/479684 18299735 PMC2659137

[8] van Wifferen F , de Jonge L, Worthington J, et al COVID-19 and Cancer Global Modelling Consortium (CCGMC) working group 2. Prioritisation of colonoscopy services in colorectal cancer screening programmes to minimise impact of COVID-19 pandemic on predicted cancer burden: a comparative modelling study. J Med Screen. 2022;29(2):72–83. 10.1177/09691413211056777 35100894 PMC9087314

[9] Larose DT , Larose CD. Discovering Knowledge in Data: an Introduction to Data Mining. vol. 4. John Wiley & Sons; 2014.

[10] Sharara N , Nolan S, Sewitch M, Martel M, Dias M, Barkun AN. Assessment of a colonoscopy triage sheet for use in a province-wide population-based colorectal screening program. Can J Gastroenterol Hepatol. 2016;2016:4712192. 10.1155/2016/4712192 27446841 PMC4947491

[11] Jain D , Goyal A, Zavala S. Predicting colonoscopy time: a quality improvement initiative. Clin Endosc. 2016;49(6):555–559. 10.5946/ce.2015.110 26996219 PMC5152781

[12] Ristikankare M , Hartikainen J, Heikkinen M, Janatuinen E, Julkunen R. The effects of gender and age on the colonoscopic examination. J Clin Gastroenterol. 2001;32(1):69–75. 10.1097/00004836-200101000-00016 11154176

[13] Devi SP , Rao KS, Sangeetha SS. Prediction of surgery times and scheduling of operation theaters in ophthalmology department. J Med Syst. 2012;36(2):415–430. 10.1007/s10916-010-9486-z 20703709

[14] Bentayeb D , Lahrichi N, Rousseau LM. Patient scheduling based on a service-time prediction model: a data-driven study for a radiotherapy center. Health Care Manag Sci. 2019;22(4):768–782. 10.1007/s10729-018-9459-1 30311107

