# Supplementary material for: Reducing endoscopic procedure backlog by improving efficiency: a predictive model and machine learning-based scheduling approach
Source: J Can Assoc Gastroenterol. 2026 Feb 5;9(2):110–5. doi: 10.1093/jcag/gwaf006 (PMC13123678; doi:10.1093/jcag/gwaf006)
Supplement: gwaf006_Supplementary_Data [file gwaf006_supplementary_data.zip › suppl_data/new data/gwaf006_AQ11_gwaf006_suppl_Supplementary_Figures_1-3_Tables_1-3 (1).docx]

**Supplementary documents**

**Supplementary Table** 1 - Distribution of patients by priority based on the Quebec provincial colonoscopy referral sheet

| Priority class | Recommended wait time | Percentage |
| --- | --- | --- |
| P2 | 2 weeks | 2*.*2% |
| P3 | 2 months | 44*.*0% |
| P4 | 6 months | 16*.*3% |
| P5 | 6 months (P4 patients have higher priority) | 13*.*2% |
| Surveillance indications | 12 months | 24*.*3% |

**Supplementary Table** 2 - Results on the test set of the different regression models.

|  | MSE | MAE | Training time (seconds) |
| --- | --- | --- | --- |
| Linear Regression | 63.86 | 5.72 | 0.43 |
| Random Forest | 63.43 | 5.66 | 0.50 |
| XGBoost | **63.09** | 5.67 | 5.64 |
| Gaussian Process | 63.85 | 5.68 | 4.74 |
| MLP | 63.75 | 5.64 | 0.59 |

**Supplementary Table 3** - Detailed results of the simulation on real data. The number of patients scheduled, the real and predicted total procedure durations in each room each day (in minutes) are reported. The time budget of each day each room was 390 minutes. Bold numbers indicate the days with overtime.

|  | fixed 30’ |  | Greedy |  |  | MIP |  |
| --- | --- | --- | --- | --- | --- | --- | --- |
| date | #patients observation | #patients | observation | prediction | #patients | observation | prediction |

Room 1

| 2019-03-18 | 13 | 396 | 13 | **396** | 380 | 13 | **409** | 387 |
| --- | --- | --- | --- | --- | --- | --- | --- | --- |
| 2019-03-19 | 13 | **408** | 13 | **408** | 377 | 14 | 379 | 382 |
| 2019-03-20 | 13 | 382 | 13 | 382 | 372 | 15 | **424** | 390 |
| 2019-03-21 | 13 | 360 | 13 | 355 | 377 | 13 | **471** | 390 |
| 2019-03-22 | 13 | **455** | 14 | **490** | 387 | 14 | **421** | 390 |
| 2019-03-23 | 13 | **397** | 13 | **397** | 387 | 13 | **418** | 388 |
| 2019-03-24 | 13 | 391 | 13 | **391** | 365 | 13 | **419** | 390 |
| 2019-03-25 | 13 | **406** | 13 | **401** | 375 | 13 | 392 | 390 |

Room 2

| 2019-03-18 | 11 | 318 | 11 | 318 | 317 | 11 | 344 | 324 |
| --- | --- | --- | --- | --- | --- | --- | --- | --- |
| 2019-03-19 | 13 | 363 | 13 | 363 | 370 | 14 | 364 | 380 |
| 2019-03-20 | 13 | **416** | 13 | **416** | 385 | 15 | **394** | 390 |
| 2019-03-21 | 13 | 383 | 13 | 378 | 374 | 14 | 385 | 386 |
| 2019-03-22 | 10 | 304 | 10 | 309 | 288 | 10 | 322 | 301 |
| 2019-03-23 | 13 | **399** | 13 | **399** | 371 | 13 | **401** | 388 |
| 2019-03-24 | 13 | **406** | 13 | **406** | 385 | 13 | **409** | 389 |
| 2019-03-25 | 13 | **434** | 13 | **434** | 375 | 13 | **453** | 390 |

**Supplementary Table 4:** Summary of Python packages and tools used for the machine learning analysis, including their versions and descriptions of their functionality.

| **Package** | **Version** | **Description** |
| --- | --- | --- |
| python | 3.8 | The core programming language used for analysis and development. |
| CPLEX | 22.1 | Optimization solver for linear programming, mixed-integer programming, and more. |
| hyperopt | 0.2.7 | Hyperparameter optimization. |
| matplotlib | 3.5.1 | Visualization library used in machine learning workflows. |
| numpy | 1.22.2 | Fundamental library for numerical computing. |
| pandas | 1.4.1 | Data analysis and manipulation library. |
| scikit-learn | 1.1.1 | Core library for machine learning algorithms and tools. |
| scipy | 1.8.0 | Scientific computing library used in machine learning tasks. |
| xgboost | 1.5.2 | Extreme Gradient Boosting library for machine learning. |

**Supplementary Figure 1: CRoss Industry Standard Process for Data Mining.**


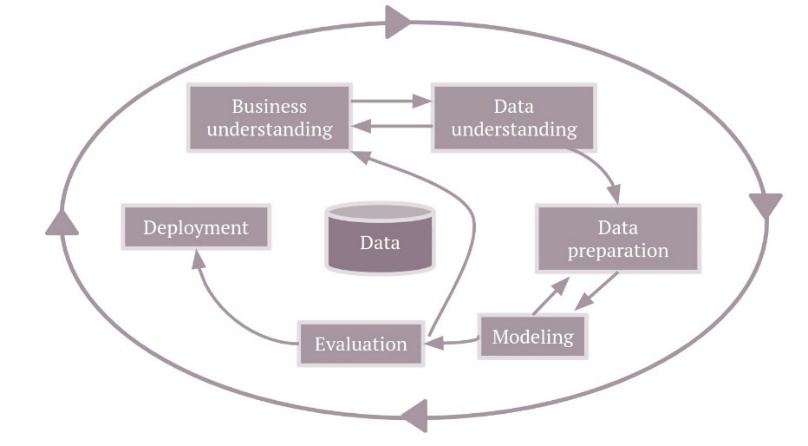


**Supplementary Figure 2: Distribution of procedure times.**


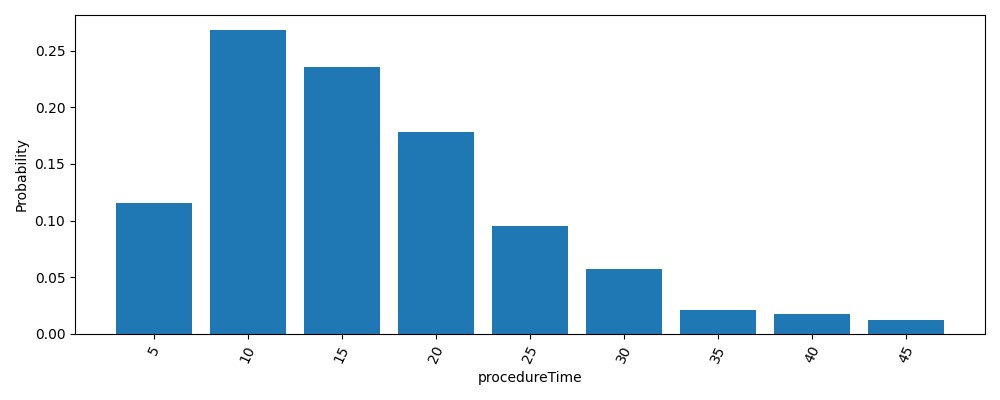


**Supplementary Figure 3: MAE of training and testing set at each step of feature selection.**


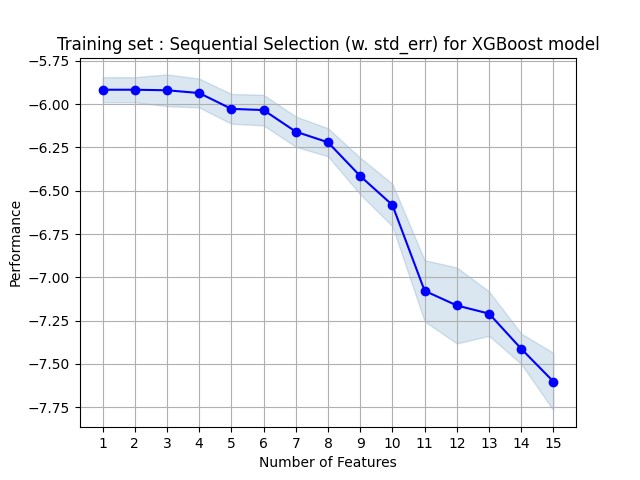


## Machine learning metrics

To evaluate the performance of our regression models, we use two classic metrics: Mean Squared Error (MSE) and Mean Absolute Error (MAE). These metrics quantify the differences between predicted (Ŷ_i_) and actual (Y_i_) values, providing insight into model accuracy.

- **Mean Squared Error** (MSE): Measures the average squared difference between predicted and actual values, calculated as:


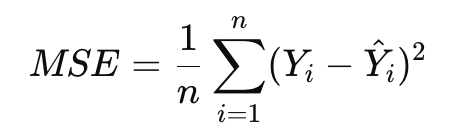


- - MSE penalizes larger errors more heavily, with lower values indicating better performance.
- **Mean Absolute Error** (MAE): Calculates the average absolute difference between predicted and actual values:


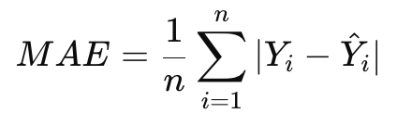


- - MAE provides an intuitive error measure in the same units as the target variable, with lower values also reflecting better performance.

## Machine learning models

This section provides a description of the machine learning models used in the project, alongside with the definition of the metrics used. In this phase, different predictive models are tested and compared.

- *Linear Regression (LR)*. LR models the relationship between a dependent variable (label) with independent variables (features) as an equation with linear terms: Y=β0 +β1 X1 +β2 X2 +...+βk Xk where Y is a dependent variable (DV), Xi an independent variables, β0 is a constant, and βi is the coefficient of Xi . The coefficients constitute a (straight or curved) line that fit the training data the best.
- *Random Forest (RF)*. RF is an ensemble learning method, which consists of multiple decision trees. Each individual tree produces a prediction. A RF ensembles those predictions and returns final decision.
- *EXtreme Gradient Boosting (XGBoost)*. XGBoost is a gradient boosted tree algorithm. It builds a model by adding new trees iteratively while using a gradient descent algorithm to minimize the loss function.
- *Gaussian Process (GP)*. GP model is a probabilistic supervised machine learning framework for regression and classification tasks. A probabilistic kernel-based model based on the assumption that the data labels are determined through a Gaussian Process of specified kernel type.
- *Multi-layer Perceptron (MLP)*. An MLP is a fully connected neural network that generates a set of output from a set of inputs. An MLP has at least one hidden layer and a specified non-linear activation function. To train the network, an MLP uses backpropagation to adjust its weighted connections using labelled data.

## Model Hyperparameter Tuning

The hyperparameter optimization for the XGBoost Regressor was performed using the Hyperopt package, leveraging Bayesian optimization via the Tree-structured Parzen Estimator (TPE). The optimization aimed to minimize the Mean Absolute Error (MAE) on a validation set. Below, we summarize the parameters, their search spaces, and the final selected values:

| Parameter | Search Space | Final Value | Description |
| --- | --- | --- | --- |
| n_estimators | [1, 200] | 42 | Number of trees in the model. |
| eta | [0, 1] | 0.89 | Learning rate for step size control. |
| gamma | [0, 1] | 0.97 | Minimum loss reduction to split a node. |
| max_depth | [1, 10] | 2 | Maximum depth of each tree. |
| min_child_weight | [1, 6] | 3 | Minimum sum of weights required in a child node. |
| subsample | [0.1, 1] | 0.51 | Fraction of samples used for training each tree. |
| colsample_bytree | [0.5, 1] | 0.83 | Fraction of features used per tree. |
| reg_alpha | [0, 1] | 0.82 | L1 regularization term. |
| reg_lambda | [0, 1] | 0.19 | L2 regularization term. |
| booster | "gbtree" | "gbtree" | Booster used for training. |
| tree_method | "exact" | "exact" | Method for tree construction. |

## Stratified results

We report the performance of the prediction model across three duration categories: short, average, and long. The number of procedures in each category is presented in Table 5. Figure 6 displays the confusion matrices generated by XGBoost using the classifications outlined in Table 5. In the confusion matrices, the diagonal cells represent correctly predicted instances, while cells above the diagonal indicate over-predictions (predicted duration > actual duration), and cells below the diagonal indicate under-predictions (predicted duration < actual duration).

For the short class (0–10 minutes), the majority of instances (58 out of 96) are over-predicted. For the average class (10–20 minutes), the model demonstrates high precision, with 76% of instances correctly predicted. However, 22.1% of instances are under-predicted, and only 1.9% are over-predicted. For the long class (20–45 minutes), most instances (88.2%) are under-predicted as belonging to the average class (10–20 minutes). These results are influenced by the imbalance in the dataset, as the long class is underrepresented, leading to reduced predictive performance for this category.

**Supplementary Table 5:** Procedure's classification by duration

| **Class** | **Duration** | **# patients** | **Percentage** |
| --- | --- | --- | --- |
| **short** | less than 10’ | 481 | 38.40% |
| **average** | 10’ to 20’ | 518 | 41.30% |
| **long** | 20’ to 45’ | 254 | 20.30% |


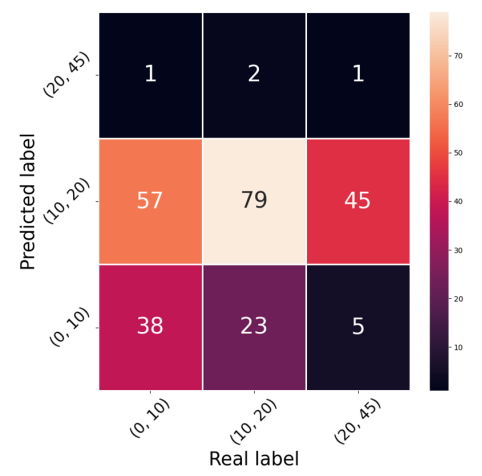


**Supplementary figure 6: Confusion matrix by procedure’s length**
